# 769 The association of a burn injury with social engagement: A Preschool-LIBRE1-5 study

**DOI:** 10.1093/jbcr/irac012.322

**Published:** 2022-03-23

**Authors:** Maree Baird, Kathleen S Skipton Romanowski, Pengsheng Ni, Khushbu F Patel, Lewis E Kazis, Colleen M Ryan, Tina L Palmieri

**Affiliations:** Shriners Hospitals for Children, Sacramento, Sacramento, California; UC Davis, Sacramento, California; Boston University School of Public Health, Boston, Massachusetts; Shriners Hospitals for Children - Boston/MGH, Boston, Massachusetts; Boston University School of Public Health, Spaulding Rehabilitation Hospital, Harvard Medical School, Boston, Massachusetts; Harvard Medical School, Boston, Massachusetts; UC Davis and Shriners Hospitals for Children Northern California, Sacramento, California

## Abstract

**Introduction:**

Social engagement, primarily through peer interactions, is an active-coping strategy that is key to community re-integration after a burn injury. For young children, social engagement encourages standard development, with burn injuries potentially affecting their likelihood to engage with others. We utilized the parent-report Preschool-LIBRE_1-5_ (Life Impact Burn Recovery Evaluation) to explore factors that can influence a child’s social engagement.

**Methods:**

Our participants were 426 parents of burned children ages one through five who completed the Preschool-LIBRE_1-5_. Four variables involving social engagement were assessed: “frequency of child avoiding other children,” “frequency of child playing alone,” “frequency of child wanting to be left alone,” and “frequency of child liking to be around other children.” Responses from a 5-point Likert scale ranging from never to always were recoded on a 3-point Likert scale such that a higher score indicated increased social engagement. A nominal logistic regression analysis was conducted with each social engagement variable as a separate dependent variable for each model. The age of the patient when the survey was completed and gender were independent variables. The models also were adjusted by the presence of a hand burn, a face burn, burn size, race, and ethnicity. A bootstrap analysis was conducted to assess internal validity of the significant findings.

**Results:**

The sample characteristics included: mean age of 3.1+1.4 years, mean time since burn injury of 1.2+1.3 years, mean total body surface area (TBSA) of 4.2+7.9, 55.2% male, and 74.2% white. Findings did not reach significance for three of the dependent variables. However, the dependent variable “frequency of child liking to be around other people” was significant with an increased odds of 35.8% for older children compared to younger ones. (OR [95% CI] =1.358 [1.027, 1.795]). Further, boys were much less likely to want to be left alone (OR [95% CI] = 2.97[1.36,6.5]). Among 500 bootstrap samples, 59% and 79% of the samples replicated the significant findings respectively.

**Conclusions:**

In preschool children with burn injuries, burn location, race, and ethnicity were not associated with the social engagement items tested in this study. Boys did show a higher likelihood of peer engagement suggesting that gender may influence a child’s desire to be alone.

